# Quality of life and coping strategies of healthcare workers during the first wave of the COVID-19 pandemic in Russia

**DOI:** 10.1192/j.eurpsy.2026.11012

**Published:** 2026-07-31

**Authors:** V. I. Rozhdestvenskiy, V. V. Titova, I. A. Gorkovaya, D. O. Ivanov, Y. S. Aleksandrovich

**Affiliations:** Department of Psychosomatics and Psychotherapy, Saint Petersburg State Pediatric Medical University, Saint Petersburg, Russian Federation

## Abstract

**Introduction:**

During the first wave of the novel coronavirus pandemic in Russia, healthcare workers were on the front line in the fight against this dangerous disease. Studies show that the quality of life of medical professionals declined during this period. It is essential to examine whether the coping strategies employed by Russian medical professionals are effective in enhancing their quality of life.

**Objectives:**

The study aimed to examine the quality of life of Russian healthcare workers during the first wave of the COVID-19 pandemic. The relationships between domains of quality of life and coping strategies were also analysed.

**Methods:**

Data were collected between May and July 2020 using a Google Form developed by the authors. The sample comprised 45 healthcare workers residing in Russia. The quality of life was assessed using the WHOQOL-BREF-26 instrument, and coping strategies were evaluated with the COPE-60 inventory. Both instruments had been previously adapted for use in the Russian context.

**Results:**

We found the following average values for the domains of quality of life: ‘physical and psychological well-being’ — M = 21.58±2.82; ‘self-perception’ — M = 21.02±2.02; ‘microsocial support’ — M = 10.96±2.20; ‘social well-being’ — M = 27.36±4.00. No statistically significant correlations were found between the domains ‘physical and psychological well-being,’ ‘microsocial support,’ and coping strategies. ‘Self-perception’ was negatively correlated with restraint (r_s_ = -0.303, p < 0.05), and ‘social well-being’ was negatively correlated with the use of ‘sedatives’ (r_s_ = -0.425, p < 0.01).

**Conclusions:**

During the first wave of the COVID-19 pandemic, the physical and psychological well-being of Russian healthcare workers, including indicators such as physical pain, need for medical care, physiological tone, transportation accessibility, and sleep, as well as their satisfaction with the immediate social environment, were not significantly associated with coping strategies. However, limiting activity and abandoning efforts to change the situation within a short time frame were linked to lower satisfaction among healthcare workers. Furthermore, the use of alcohol and psychoactive substances was associated with their social well-being.

**Disclosure of Interest:**

None Declared

